# Idiopathic epidural lipomatosis of the lumbar spine

**DOI:** 10.11604/pamj.2017.28.156.13991

**Published:** 2017-10-19

**Authors:** Mohamed Badri, Dhia Kaffel

**Affiliations:** 1 El Manar, Tunis University, Faculty of Medicine of Tunis, Department of Neurosurgery, Burns and Trauma Center, Ben Arous, Tunisia; 2 Rhumatology Department, Kassab Institute, Manouba, Tunisia

**Keywords:** Epidural lipomatosis, lumbar spine, MRI

## Image in medicine

A 50-year-old male without medical history presented with progressive development of motor weakness in his lower extremities and urinary leakage. For one year, he had experienced severe lower back pain associated to bilateral radicular claudication of the lower limbs. Physical examination revealed stepping with bilateral foot dorsiflexor weakness (2/5). Deep-tendon reflexes were abolished in both lower limbs with hypoesthesia of the lower left extremity. Examination of Upper limbs was normal and the body mass index was 37 kg/m^2^. Magnetic resonance imaging (MRI) of the lumbar spine showed a multilevel high intensity epidural mass lesion compressing the dural sac extending from L1 to L5 level, on T1 and T2 weighted images (WI) (A & B). Axial MRI T2-WI showing a hyperintense anterior epidural mass with deformation of the dural sac at the level of L4 (C). Frontal MRI myelography showed a complete stop of the cerebro-spinal fluid at lumber levels (D). The patient was operated. Laminectomy and epidural decompression at L3, L4 and L5 levels with removal of epidural fat has been performed. Pathologic examination revealed nodular proliferation of mature fat cells, with lipomatosis. Postoperative examination showed improvement of clinical signs.

**Figure 1:**
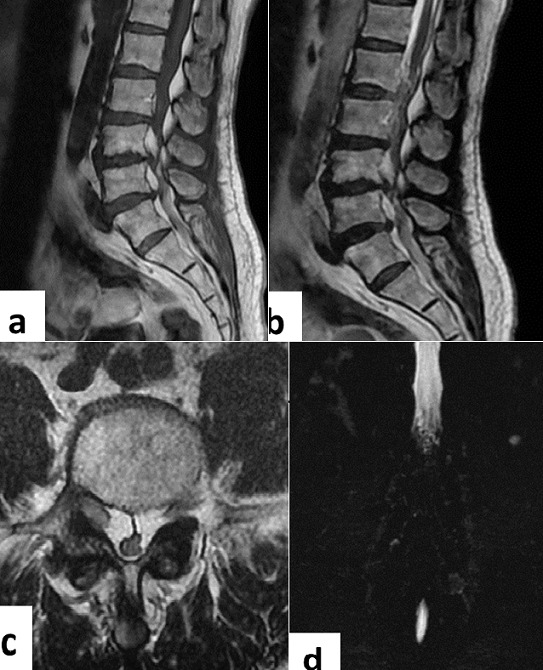
(A) sagittal T1 weighted images MRI shows posterior and anterior epidural adipose tissue compressing the dural sac extending from L1 to L5 segment with high signal intensity; (B) sagittal T2 weighted images MRI featured multilevel high intensity epidural mass lesion compressing the dural sac; (C) axial T2 weighted images MRI noticed a hyperintense anterior epidural mass with deformation of the dural sac; (D) frontal MRI myelography shows complete stop of the cerebro-spinal fluid on the lumbar spine

